# Anti-Amyloidogenic and Cyclooxygenase Inhibitory Activity of *Guettarda speciosa*

**DOI:** 10.3390/molecules24224112

**Published:** 2019-11-14

**Authors:** Mario A. Tan, Mark Wilson D. Lagamayo, Grecebio Jonathan D. Alejandro, Seong Soo A. An

**Affiliations:** 1 Department of Bionano Technology, Bionano Research Institute, Gachon University, 1342 Sungnam-daero, Sujung-gu, Seongnam-si, Gyeonggi-do 461-701, Korea; 2 Graduate School, University of Santo Tomas, Manila 1015, Philippines; mwdlagamayo@gmail.com (M.W.D.L.); gdalejandro@ust.edu.ph (G.J.D.A.); 3 Research Center for the Natural and Applied Sciences, University of Santo Tomas, Manila 1015, Philippines; 4 College of Science, University of Santo Tomas, Manila 1015, Philippines

**Keywords:** Alzheimer’s disease, COX-1, cytotoxicity, *Guettarda speciosa*, thioflavin T

## Abstract

*Guettarda speciosa* is known in traditional folk medicine for treating cough, cold, sore throat, fever, wounds, epilepsy, and headaches. To discover the scientific pharmacological potential of *G. speciosa*, we explore its anti-inflammatory, cytotoxicity, and inhibition of amyloid-*beta* (Aβ) aggregation effects. Cyclooxygenase assay of the *G. speciosa* CHCl_3_ (GSC) extract and *G. speciosa* MeOH (GSM) extract are more selective to COX-1 inhibition with a 50% inhibitory concentration (IC_50_) of 3.56 μg/mL for the GSC extract and 4.98 μg/mL for the GSM extract. Neuroblastoma SH-SY5Y inhibition and thioflavin T assay amyloid-*beta* (Aβ) aggregate inhibition of the GSM and GSC extracts showed their potential therapeutic effects against Alzheimer’s disease. The putative compounds from the LC-MS analysis could be responsible for the observed activities. The results suggest that *G. speciosa* possesses anti-inflammatory and anti-neurodegenerative properties and a promising lead as a source of pharmacologically active compounds.

## 1. Introduction

*Guettarda speciosa* L. (Rubiaceae) is a perennial shrub or small tree, which grows in coastal habitats in tropical areas. This species is the only representative of the genus *Guettarda* L. in the Philippines [1]. The genus is widely distributed from East Africa to South and Southeast Asia and the South Pacific [2]. It is regarded as a medicinal plant used in traditional folk medicine for treating postpartum infection, cough, cold, sore throat, dysentery, fever, boils, wounds, epilepsy, and headache [3,4,5,6]. In African medicinal plants, the flower decoction was combined with *Ocimum americanum* L. and *O. gratissimum* L. to treat malaria, while the roots are used for diarrhea (decoction), rheumatism (rubdown on articulations), and pelvic pain (massage) [7]. These traditional folkloric claims were corroborated by pharmacological studies including the antiepileptic activity of the inner bark extract from India [5] and the anti-inflammatory activity in murine macrophages of the methanolic extract from Indonesia [8]. Phytochemical analysis has elaborated the presence of iridoids and their glucosides, phenolics, glycerol derivatives, steroids, triterpenoids [9,10], and fatty acids [11]. There is limited information on the biological activities and chemical constituents associated with *G. speciosa*.

To address this gap, and in the interest of searching for medicinal Rubiaceae plants from the Philippines with potential anti-inflammatory and anti-neurodegenerative activities [12,13,14,15], we herein describe the acute toxicity, cyclooxygenase inhibition, and anti-amyloidogenic activity of the extracts of *G. speciosa*.

## 2. Results

### 2.1. Acute Oral Toxicity

**Figure 1:**
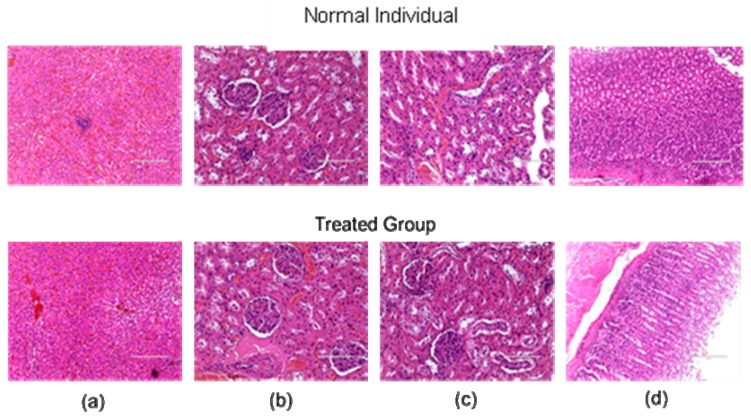
Histopathological examination of (**a**) liver, (**b**) left kidney, (**c**) right kidney, and (**d**) stomach in normal and *G. speciosa* extract-treated (GSM) groups. No significant changes were observed in the examined vital organs of the GSM-treated groups when compared to the normal control group.

No death was observed among the animals over the 14-day period. Hence, assessment of the acute oral toxicity indicated that the *G. speciosa* MeOH (GSM) extract was safe and nontoxic up to 2000 mg/kg following the Organization for Economic Co-operation and Development (OECD) 425 guidelines. Prominent signs of toxicity and abnormalities were also not observed. Moreover, the post toxicity and gross necropsy study showed that all vital organs, such as the liver, kidneys, and stomach, were comparable to the control group as indicated in the histopathological results (Figure 1) analyzed by a licensed veterinarian.

### 2.2. COX-1 and COX-2 Assay

Aerial parts of *G. speciosa* were extracted with MeOH to afford the crude extract (GSM).Various extracts of *G. speciosa* with varying polarity were prepared using solvent partitioning. Nonpolar compounds are contained in the *G. speciosa* hexane (GSH) extract, semi-polar compounds in the *G. speciosa* CHCl_3_ (GSC) extract, and polar compounds in the *G. speciosa* aqueous (GSA) extract. All the extracts were dried free of solvents prior to use in the succeeding experiments.

**Figure 2:**
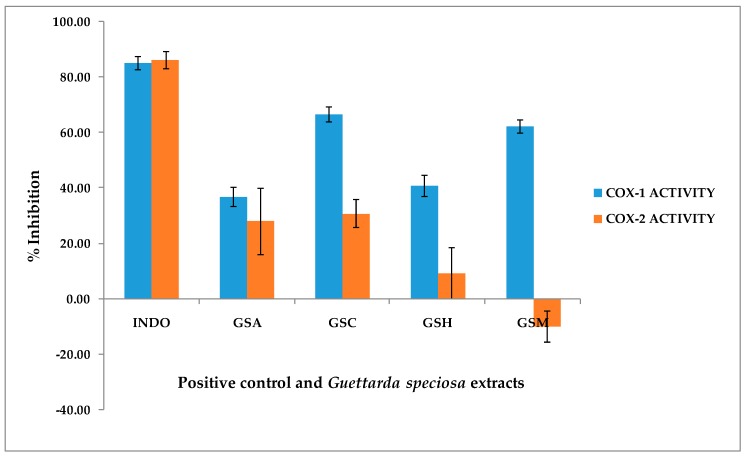
In vitro cyclooxygenase screening of *Guettarda speciosa* (*G. speciosa*) extracts at a concentration of 10 μg/mL. The *G. speciosa* extracts exhibited an inhibition to the COX-1 enzyme with the chloroform GSC and methanol GSM extracts showing a greater than 50% inhibition. Indomethacin (4.0 mM) (INDO) was used as the positive control. GSA—*G. speciosa* aqueous extract; GSC—*G. speciosa* CHCl_3_ extract; GSH—*G. speciosa* hexane extract; GSM—*G. speciosa* MeOH extract.

The cyclooxygenase screening assay results of the *G. speciosa* extracts (Figure 2) showed a greater inhibition to the COX-1 enzyme as compared to the COX-2. The extracts were initially tested at a concentration of 10 μg/mL. More than 50% inhibition for the COX-1 enzyme was observed for GSC (66.68% ± 2.77) and GSM (62.25% ± 2.39) extracts. None of the extracts gave a 50% inhibition using COX-2 enzyme with the GSC extract exhibiting the highest inhibition with 30.85% ± 5.11. Interestingly, the GSM extract exhibited a negative inhibition with COX-2 (−9.98% ± 5.62). The percentage inhibition of the extracts had a significant difference when compared to the positive control, indomethacin (4.0 mM) (85.1–86.3%), at *p* < 0.05.

Based on the screening results, the 50% inhibitive concentration (IC_50_) of the GSC and GSM extracts for COX-1 were also determined using seven concentrations (0.5, 1, 5, 10, 40, 70, and 100 µg/mL). The results indicate an IC_50_ of 3.56 μg/mL for the GSC extract and 4.98 μg/mL for the GSM extract. Because of the promising results in the cyclooxygenase assay, the GSM and GSC extracts were further evaluated via cell viability and thioflavin T assays.

### 2.3. Cell Viability

**Figure 3:**
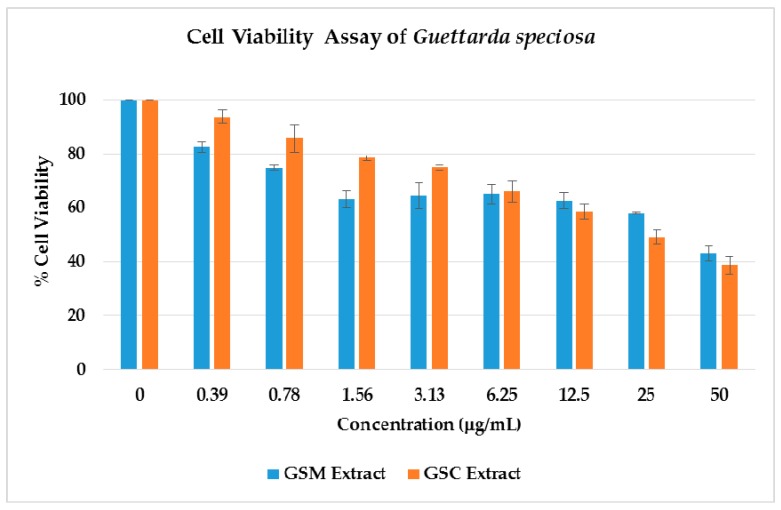
Effect of *Guettarda speciosa* MeOH (GSM) and CHCl_3_ (GSC) extracts on cell viability in neuroblastoma SH-SY5Y cells. Cell viability was determined using the ATP luminescence assay. The results indicate % cell viability vs. the negative control (mean ± SD of triplicate measurement). All of the extracts exhibited a significant difference on the % cell viability on the negative control vs. the plant extracts at *p* < 0.05, except for the GSC extract at 0.39 μg/mL).

The cytotoxicity of the GSM and GSC extracts was explored against neuroblastoma SH-SY5Y utilizing the ATP luminescence assay. The ATP serves as the cell’s most important chemical energy storage for all biological processes. When cells are exposed to environmental or metabolic stressors (depleted iron and carbon sources), their ability to produce ATP is impeded. Hence, cellular ATP measurement is a good indication of a cell’s metabolic health and subsequent viability. As illustrated in Figure 3, the GSM extract exhibited a 17% cell growth inhibition using the smallest concentration of 0.39 μg/mL, followed by 25% growth inhibition at 0.78 μg/mL. Surprisingly, an almost similar trend was observed, using 12.5, 6.25, 3.13, and 1.56 μg/mL concentrations with cell growth inhibitions at 35–38%. The highest concentration at 50 μg/mL showed a cell growth inhibition of 57%. Compared to the negative control, a significant difference was shown on the cell cytotoxicity of the extracts at all the given concentrations (*p* < 0.05). Cytotoxicity was also observed on the GSC extracts in a concentration-dependent manner from 0.39 to 50 μg/mL as observed in Figure 3. A 62% cell growth inhibition was noted on the 50 μg/mL, followed by the 25 μg/mL with 50% inhibition. The lowest concentration, 0.39 μg/mL, showed only a 6% cell growth inhibition with a cell viability of 94%. Statistical analyses also indicated a significant difference on the percentage cell viability of the GSC extracts when compared to the control (100% cell viability), with the exception of the lowest concentration (0.39 μg/mL).

Through the cell viability ATP luminescence assay, the half maximal inhibitory concentration of the cell population death was determined. The GSM extract inhibited the growth of SH-SY5Y cells with an IC_50_ value of 43.44 μg/mL. The GSC extract was found to be more active with an IC_50_ of 8.049 μg/mL. SH-SY5Y cells have been used in many neurological studies, such as Parkinson’s disease (PD), Alzheimer’s disease (AD), and traumatic brain injury (TBI) [16]. The cell viability of the neuroblastoma SH-SY5Y cells against the GSM extracts may suggest promising leads with respect to the identification of potential bioactive secondary metabolites in neurological diseases.

### 2.4. Thioflavin T (ThT) Assay

The abnormal aggregation of β-amyloid (Aβ), tau protein accumulation, decreased acetylcholine, oxidative stress, and neuroinflammation of the nervous system are some of the pathological hallmarks associated with Alzheimer’s disease. To address whether the GSM and GSC extracts are capable of inhibiting the aggregation of Aβ, we also utilized thioflavin T (ThT) assay using phenol red as the positive control (Table 1). The GSC extract at 50 μg/mL exhibited the highest inhibition of the Aβ fibril formation with 65.78%. This is also statistically comparable (*p* < 0.05) to the inhibition of the positive control at 69.85%. The GSM extract gave a 54.71% inhibition but is statistically different from the positive control (*p* < 0.05). The observed activity of the GSC and GSM extracts may be attributed to the presence of secondary metabolites, which could be capable of inhibiting the Aβ fibril formation.

### 2.5. Metabolite Profiling

**Figure 4:**
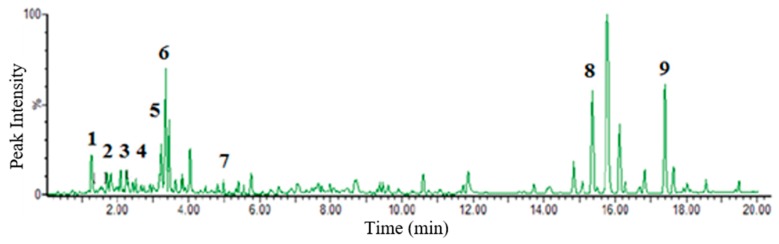
Chromatogram of *G. speciosa* MeOH (GSM) extract. Nine putative compounds were identified utilizing the UNIFI data analysis software and comparing the acquired MS spectra to library matching using the Traditional Chinese Medicine (TCM) library that is incorporated in the UNIFI analysis software. All of these compounds were previously isolated from other *Guettarda* species. The x-axis is the retention time in minutes, while the y-axis is the peak % signal intensity.

**Figure 5:**
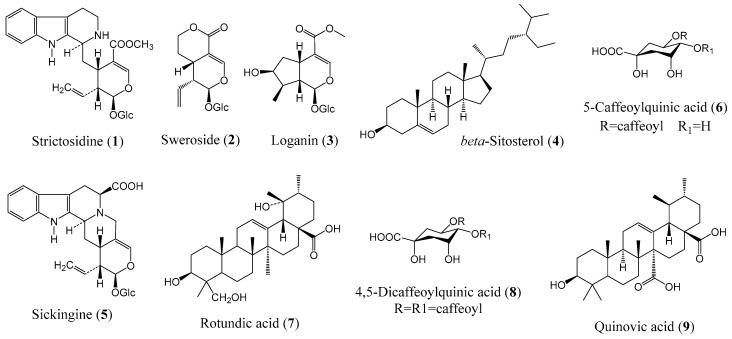
Structures of putatively identified compounds from *G. speciosa*.

A total of nine putatively identified compounds were determined from the GSM extract using LC-MS analysis. Optimized run method produced the LC chromatogram as shown in Figure 4, where the Y-axis represents the % signal intensity and the X-axis is the retention time in minutes. The compounds presented (Table 2 and Figure 5) are those that fall within the “good match” standards of the Traditional Chinese Medicine (TCM) library [17,18]. These putatively identified nine compounds were also previously isolated from the different *Guettarda* species based on the available literature data. Loganin (**3**) was isolated from *G. platypoda* DC. [19] and *G. pohliana* Müll. Arg. [20]; rotundic acid (**7**) from *G. angelica* Mart. ex Müll. Arg. [21] and *G. platypoda* [22]; quinovic acid (**9**) from *G. angelica* [23] and *G. platypoda* [22,24]; strictosidine (**1**), sickingine (**5**), 5-caffeoylquinic acid (**6**), and 4,5-dicaffeoylquinic acid (**8**) from *G. acreana* K. Krause [3]; sweroside (**2**) from *G. platypoda* [24] and *G. pohliana* [20]; and β-sitosterol (**4**) from *G. platypoda* [22]. Interestingly, the putatively identified compounds exhibit diverse biological activities as reported in the literature (Table 2). The putatively identified metabolites with their reported activities in the literature may explain the biological activities exhibited by the extracts in this study. Several compounds were identified to have anti-inflammatory activity (**2**, **4**, and **6**), cytotoxicity (**2** and **3**), and neuroprotective (**3**) activity.

## 3. Discussion.

In our continuous study using endemic Rubiaceae species indigenous to the Philippines, we described the potential therapeutic effects of *G. speciosa* extracts. The leaf extracts of *G. speciosa*, free from any trace of solvents, have been shown to inhibit *in vitro* the aggregation of Aβ_1–42_, cyclooxygenase-1 enzyme, and cell growth of neuroblastoma SH-SY5Y cells. These biological activities may be ascribed to the compounds that were characterized using untargeted LC-MS. Natural products from plants continue to be the source of pharmacologically active compounds with diverse structures in the treatment or prevention of various diseases. In this experiment, these compounds were defined as indole alkaloids (**1**, **5**), iridoids (**2**, **3**), steroid (**4**), triterpenoids (**7**, **9**), and polyhydroxy cyclic acids (**6**, **8**). Pharmacological studies on *G. platypoda* have reported the synergistic action of quinic acid glycosides with β-sitosterol and triterpenes with anti-inflammatory activity, while the mechanism of β-sitosterol is comparable to hydrocortisone [32]. Most of the Rubiaceae species also contained iridoids and indole alkaloids [40], which are known to possess anti-inflammatory activities [41,42].

This study also described the inclination of the *G. speciosa* extracts to COX-1 inhibition as compared to the COX-2 enzyme. Both COX-1 and COX-2 perform a complex function in the mechanism of central nervous system (CNS) inflammation [43]. Most studies prefer a COX-2 inhibition, because COX-1 is involved in the cytoprotective function in the gastrointestinal system. Moreover, the suppression of COX-1 can result in side effects, including ulcers and bleeding [44]. Several studies have reconsidered the advantages of selective COX-1 inhibition. As stated, COX-1-dependent prostaglandin synthesis is implicated in pathological progressions, including atherosclerosis, cancer, endothelial dysfunction, neuroinflammation, preterm labor, and pain [43,45].

Alzheimer’s disease (AD) is the most common cause of neurodegenerative dementia in elderly people, often associated witha progressive memory loss and other cognitive impairments [12]. Abnormal β-amyloid (Aβ) deposition, tau protein aggregation, a decreased level of acetylcholine, oxidative stress, and neuroinflammation of the nervous system are numerous causes associated with enhanced AD progression [46]. Although there is no cure for AD, currently, only five compounds (donezipil, tacrine, rivastigmine, galantamine, and memantine) are available and approved in the market to reduce the symptoms associated with AD [47]. The biological activity of the GSM extracts to inhibit the Aβ aggregation exhibits the potential of this medicinal plant as a new pharmacologicallyactive material or therapeutic agent to minimize the effect of AD. To the best of our knowledge, this is the first report on the SH-SY5Y cytotoxicity, Aβ aggregation prevention, and COX-1 inhibition activities associated with *G. speciosa*.

## 4. Materials and Methods

### 4.1. PlantMaterials

Fresh leaves of *G. speciosa* were collected from Bantayan Island, Cebu, Philippines (11°12’60.00” N, 123°43’59.99” E) in April 2017. The plant was collected and identified by Grecebio Jonathan Alejandro, a Philippine Rubiaceae specialist. A voucher specimen was kept at the University of Santo Tomas Herbarium (USTH 014369).

### 4.2. Extraction and Fractionation of Extracts

Air-dried, ground leaves of *G. speciosa* (1.7 kg) were placed in a percolator and extracted with MeOH. The ground leaves were allowed to soak overnight. The extract was drained, collected, and concentrated under reduced pressure using a rotary evaporator. A total of 15.0 L MeOH was used with the extraction process repeated thrice. The procedure obtained 207 g of the MeOH extract (GSM), and a portion (158 g) was suspended in distilled H_2_O (300 mL) and partitioned exhaustively with hexane (2500 mL). The combined hexane layer was dried with anhydrous Na_2_SO_4_ and concentrated under reduced pressure to obtain the hexane extract (GSH, 23.2 g). The aqueous layer was partitioned exhaustively with CHCl_3_ (2800 mL). The collected CHCl_3_ layer was dried with anhydrous Na_2_SO_4_ and concentrated under reduced pressure to obtain the CHCl_3_ extract (GSC, 10.7 g). The aqueous layer was freeze-dried using a lyophilizer (Thermo Fisher Scientific, Singapore) to obtain the aqueous extract (GSA, 18.7 g).

### 4.3. Animal Study

The experiment protocol (UST-IACUC code number RC2017-890915) was approved by the Institutional Animal Care and Use Committee (IACUC) at the Research Center for Natural and Applied Sciences (RCNAS), University of Santo Tomas (UST), and was further issued by the Philippine Bureau of Animal Industry and Animal Research Permit.

Six female Sprague-Dawley rats were used to assess the acute oral toxicity following the OECD 425 guidelines. The animals were housed in the UST-RCNAS Animal House and acclimatized to laboratory conditions for seven days before conducting the experiment. They were fed with standard rodent pellets and given access to clean drinking water. The laboratory conditions were maintained at a temperature of 25 ± 3 °C, humidity at 60 ± 4%, and a 12/12 h light/dark cycle.

### 4.4. Acute Oral Toxicity (OECD 425 Guidelines)

All animals were fasted for 24 h to determine their actual weight before receiving the crude extract. One rat served as a normal control group and was treated only with water as the vehicle medium. The animals were given 2000 mg/kg GSM via gastric gavage. The animals were observed periodically for 24 h for signs of toxicity; thereafter, they were observed daily for 14 days. The animals were then sacrificed by CO_2_ inhalation. Gross necropsy, observation of gross pathological changes, and microscopic examination of all livers, kidneys, and stomachs of the test animals were performed by a licensed veterinarian.

### 4.5. COX-1 and COX-2 Assay

The cyclooxygenase assay was based on a previous protocol [15] as follows. The following were added to 150 μL of 100 mM Tris: 10 µL of 10 ppm plant extracts in DMSO, 10 µL of 1000 µM Hemin, and 10 µL of 250 U/mL COX-2 or COX-1 enzyme (Cayman Chemicals, Singapore). Indomethacin (Sigma Aldrich, St. Louis, MO, USA) was used as the positive control, and DMSO served as the negative control. The mixture was incubated at 25 °C for 15 min. After incubation, 10 µL of 200 µM amplex red was added to the mixture. Then, 10 µL of 2000 µM arachidonic acid (Sigma Aldrich, St. Louis, MO, USA) was added, and the reaction fluorescence absorbance was monitored for 2 min using Varioskan Flash (Thermo Scientific, Waltham, MA, USA) with excitation and emission wavelengths at 535 and 590 nm, respectively. The percent inhibition of the samples and the positive control were determined based on the averaged slope of each replicate using the following formula:

% Inhibition = [(Slope uninhibited − Slope inhibited)/Slope uninhibited] ∗ 100.(1)

“Slope uninhibited” is the slope of the line from the fluorescence intensity vs. time plot of the negative control group, and “slope inhibited” is the slope of the line from the fluorescence intensity vs. time plot of the samples/positive control. The method above was also done for the GSM and GSC extracts at different effective well concentrations (0.5, 1, 5, 10, 40, 70, and 100 μg/mL) to obtain the IC_50_ in μg/mL. Three trials consisting of three replicates per trial were done for each concentration of each sample.

### 4.6. Cell Viability Assay

The neuroblastoma cells (SH-SY5Y) were purchased from the American Type Culture Collection (ATCC, Manassas, VA, USA). SH-SY5Y cells were maintained in Dulbecco’s modified eagle media (DMEM) supplemented with 10% fetal bovine serum (FBS), 1% kanamycin, and 1% penicillin. Cell cultures were maintained at 37 °C in 5% CO_2_ and passaged once per week. The SH-SY5Y cells were subcultured into a 96-well plate at 2 × 10^5^ cells/well and incubated for 24 h. After incubation, the cells were treated with plant extracts at different concentrations and incubated for another 72 h. The medium was removed, and the wells were washed with phosphate-buffered saline (PBS). Fresh medium (100 μL) was added and incubated for another 30 min. After incubation, CellTiter-Glo^®^ luminescent reagent (100 μL; Promega, Madison, WI, USA) was added, and the luminescence was measured using a PerkinElmer Victor-3^®^ multi-plate reader (PerkinElmer, Waltham, MA, USA) [48]. The 50% inhibitive concentrations of the plant extracts were calculated using a nonlinear regression curve fit (GraphPad Prism ver. 6, San Diego, CA, USA). The values representing cell viability were expressed as means ± standard deviation (SD) of three trial experiments.

### 4.7. Thioflavin T (ThT) Fluorescense Assay

Aβ_1-42_ (Aggresure™)(10 μM, 30 μL) in PBS (pH 7.4) was incubated with (20 μL) or without the tested extracts at 37 °C for 24 h in a 384-well plate. Then, 20 μL ThT solution (50 μM) in glycine-NaOH buffer (pH 9) was added. The fluorescence signal was measured (excitation wavelength, 450 nm; emission wavelength, 510 nm) using a PerkinElmer Victor-3^®^ multi-plate reader. The percentage of aggregation inhibition was calculated using the following equation: [(1-*I*_Fi_/*I*_Fc_) ∗ 100%], where *I*_Fi_ and *I*_Fc_ are the fluorescence absorbance with and without the inhibitors, respectively, after subtracting the background fluorescence of the ThT solution [46].

### 4.8. Untargeted LC-MS Metabolite Profiling

Untargeted LC–MS/MS analysis of the GSM extract was performed using a Xevo G2-S Qtof (Waters Corp., Singapore). The separation was achieved using a BEH HSS T3 column (50 × 2.1 mm internal diameter). The system delivered a constant flow of 0.4 mL/min, and the mobile phase consisted of 5% CH_3_CN and 0.1% HCOOH. The injection volume was 1 µL. For operation in MS/MS mode, a mass spectrometer with an electrospray interface (ESI) was used, and the parameters were set as follows: capillary voltage, 3.0 kV for negative mode; source temperature, 120 °C; desolvation temperature, 400 °C; cone gas flow, 100 L/h; and desolvation gas flow, 1000 L/h. Low collision energy at 6 V, high collision energy at 20–50 V, and lock mass solution at 1 ng/µL were used to calibrate mass accuracy. All LC–MS/MS data were processed by the MassLynx version 3.5 NT Quattro data acquisition software (Milford, MA, USA). For putative compound identification, accurate mass screening was carried out using the UNIFI data analysis software (San Mateo, CA, USA). The acquired MS spectra were subjected to library matching using the Traditional Chinese Medicine (TCM) library that is integrated within the UNIFI analysis software. Annotation of the candidate masses was based on the accurate mass match, isotopic ratio match, and precursor ion intensity counts. The criteria for a component ID to be considered a good match were as follows: a mass accuracy error ≤ 5 mDa or ≥ −5 mDa and a response precursor for precursor ion ≥2000.

### 4.9. Statistical Analysis

All values were reported as mean values with standard deviations (mean ± SD). Statistical significance of the data was analyzed by one-way ANOVA and Levene’s test followed by Tukey’s honestly significant difference (HSD) test (GraphPad Prism 5 software package, version 5.02, GraphPad Software Inc., San Diego, CA, USA), and *p* < 0.05 was considered statistically significant.

## 5. Conclusions

This study has demonstrated the first therapeutic potential of *G. speciosa* on neuroblastoma cytotoxicity, cyclooxygenase-1 inhibition, and the control of Aβ aggregation. The results of the untargeted LC-MS metabolite profiling also describe several compounds, which might be pharmacologically relevant. Hence, deeper understanding of the chemistry and pharmacological aspect of *G. speciosa* is warranted as this plant is being utilized in traditional folk medicine. It also presents its significance as a prospective biologically active material for further development of novel and safer plant-based agents and/or pharmacologically relevant natural products for anti-inflammatory or anti-neurodegenerative diseases.

## Figures and Tables

**Table 1:** Thioflavin T (ThT) assay results of *Guettarda speciosa* extracts.

	Aβ_1-42_ Aggregation Inhibition (%) ^a^
Phenol Red (50 μM) ^b^	69.85 ± 0.29
GSM Extract (50 μg/mL)	54.71 ± 2.92
GSM Extract (5 μg/mL)	17.20 ± 0.85
GSC Extract (50 μg/mL)	65.78 ± 3.12 *
GSC Extract (5 μg/mL)	24.63 ± 1.19

^a^ The values are expressed as mean ± SD of three trial experiments. ^b^ The positive control. * No significant difference with the positive control at *p* < 0.05. GSM—*G. speciosa* MeOH extract; GSC—*G. speciosa* CHCl_3_ extract.

**Table 2:** LC-MS putative compounds from *G. speciosa* (GSM) extract.

RT	Exact Mass		Elemental Composition	Error (ppm)	Putative Identity	Biological Activity
	Calculated	Observed				
1.27	530.22644	530.22500	C_27_H_34_N_2_O_9_	−2.63	Strictosidine (1)	Antimicrobial [25]
2.26	358.12638	358.12690	C_16_H_22_O_9_	1.45	Sweroside (2)	Cytotoxic [26]; Antigenotoxic [27]; Antiosteoporotic [28]; Anti-inflammatory [29]
2.35	390.15259	390.15900	C_17_H_26_O_10_	2.35	Loganin (3)	Antigenotoxic [27]; Neuroprotective [30]; Cytotoxic [31]
2.42	414.38617	414.38650	C_29_H_50_O	0.72	β-Sitosterol (4)	Anti-inflammatory [32,33]; Antipyretic [33] Anthelminthic, Antimutagenic, Analgesic [34]
3.25	528.21100	528.21050	C_27_H_32_N_2_O_9_	−0.94	Sickingine (5)	
3.37	354.09509	354.09430	C_16_H_18_O_9_	−2.25	5-Caffeoylquinic acid (6)	Antimicrobial [35] Anti-inflammatory [36]
4.93	488.35019	488.35210	C_30_H_48_O_5_	3.88	Rotundic acid (7)	Antimicrobial [37,38]
15.37	516.45500	516.45460	C_25_H_24_O_12_	−0.77	4,5-Dicaffeoylquinic acid (8)	Antipigmentation [39]
17.35	486.33453	486.33290	C_30_H_46_O_5_	−3.28	Quinovic acid (9)	

RT—retention time in minutes.
